# A ChIP-Seq Benchmark Shows That Sequence Conservation Mainly Improves Detection of Strong Transcription Factor Binding Sites

**DOI:** 10.1371/journal.pone.0018430

**Published:** 2011-04-14

**Authors:** Tony Håndstad, Morten Beck Rye, Finn Drabløs, Pål Sætrom

**Affiliations:** 1 Department of Cancer Research and Molecular Medicine, Norwegian University of Science and Technology, Trondheim, Norway; 2 Department of Computer and Information Science, Norwegian University of Science and Technology, Trondheim, Norway; University of Pennsylvania, United States of America

## Abstract

**Background:**

Transcription factors are important controllers of gene expression and mapping transcription factor binding sites (TFBS) is key to inferring transcription factor regulatory networks. Several methods for predicting TFBS exist, but there are no standard genome-wide datasets on which to assess the performance of these prediction methods. Also, it is believed that information about sequence conservation across different genomes can generally improve accuracy of motif-based predictors, but it is not clear under what circumstances use of conservation is most beneficial.

**Results:**

Here we use published ChIP-seq data and an improved peak detection method to create comprehensive benchmark datasets for prediction methods which use known descriptors or binding motifs to detect TFBS in genomic sequences. We use this benchmark to assess the performance of five different prediction methods and find that the methods that use information about sequence conservation generally perform better than simpler motif-scanning methods. The difference is greater on high-affinity peaks and when using short and information-poor motifs. However, if the motifs are specific and information-rich, we find that simple motif-scanning methods can perform better than conservation-based methods.

**Conclusions:**

Our benchmark provides a comprehensive test that can be used to rank the relative performance of transcription factor binding site prediction methods. Moreover, our results show that, contrary to previous reports, sequence conservation is better suited for predicting strong than weak transcription factor binding sites.

## Introduction

A classical but still unsolved problem in the field of bioinformatics is to predict the genomic loci of transcription factor binding sites (TFBS). The mapping of TFBS is important to infer the regulatory networks of transcription factors (TF) which are key controllers of gene expression. Experimental and computational techniques are interdependent [1], and since traditional experimental techniques for mapping TFBS can be laborious and new high-throughput methods such as ChIP-seq are not readily available or effective in all cell contexts [2], computational prediction of binding sites is still a highly active area of research in bioinformatics.

Most prediction methods are based on searching for known sequence motifs, and though many different approaches have been investigated to improve the apparent low specificity of predictions [3], there is still a lack of a common reference dataset on which to judge and compare a method's prediction performance. While benchmarking studies have been done for the related problem of motif *discovery* [4]–[6], we are not aware of any attempts at creating a benchmark for the motif *search* problem. Most methods have therefore reported results on different, synthetic or somewhat small datasets.

Chromatin immunoprecipitation followed by massively parallel DNA sequencing (ChIP-seq) is a recent high-throughput technique which can be used to map TFBS on a genome-wide scale [2]. The technique has increased the available data on possible binding sites enormously, and raised the opportunity of better evaluating the prediction accuracy of the computational prediction methods.

The purpose of this study is two-fold. First, we create a common benchmark for TFBS search methods, based on a large set of publicly available human ChIP-seq data and explore the challenges in doing so. Our focus for the benchmark is methods which search for TFBS using known models of binding sites, not ab initio TFBS discovery. Second, we test this benchmark on a small set of methods to investigate the effects of using an alternative to the common position-weight matrix (PWM) motif representation, and of using sequence conservation across related genomes to improve accuracy.

Traditionally, one of the approaches to improving TFBS prediction accuracy has been to enhance the sequence motif model with the goal of relaxing some of the constraints and assumptions of the de facto standard PWM model, such as the assumption that nucleotide positions are independent. Recently, protein binding microarray experiments have shown that the sequence variety and position-interdependence between bases in sequence motifs are even higher than previously expected, and that TFs bind a rich spectrum of k-mers not fully captured even by multiple PWMs [7]. MotifScan [8] is in this respect an interesting alternative algorithm for scoring sequence motifs as it might be better at scoring motifs where the k-mer sequences of the motif can be clustered into several highly different subclusters. Whereas a PWM approach packs all motif k-mers into a common sequence distribution model and compares a candidate k-mer to this model as a whole, MotifScan compares a candidate k-mer to the specific k-mers in the motif in a nearest-neighbor approach. We expect this to be an improvement over PWM scanning, and test both PWM scanning and MotifScan with our benchmark.

Another approach to improving TFBS prediction accuracy has been to incorporate information about sequence conservation. It has been shown that the sequence in functional regulatory regions of the DNA is more conserved than the surrounding non-functional regions [9]. By using the genomic sequence of several related genomes, it is possible to infer how conserved a potential binding sites is, and thus to have a higher confidence in the conserved predicted sites versus the non-conserved sites [3].

A particular successful example used genomes of 12 *Drosophila* species [10] with a scoring scheme which measures the total branch length in the phylogenetic subtree covering the genomes that harbor the motif. In their “MotifMap” article [11], Xie et al. test this approach using a multiple alignment of 18 placental mammals and further improve it by considering the uncertainty of the motif occurrences when calculating the branch length. Their “Bayesian branch length score” (BBLS) method is one of the latest algorithms for conserved TFBS search and is tested here with our benchmark. We also test a simpler method based on sequence conservation, which we name “Weighted sum” (WS), where we simply sum a weighted average of the motif score in the mouse and rat genome in addition to human. The WS method can be thought of as a baseline conservation-based method, and serves to illustrate how much performance can be gained with a basic conservation scheme versus a more refined approach such as the bayesian branch length score.

Although “phylogenetic footprinting” has the potential to filter out non-conserved sites and increase specificity, not all functional sites are conserved [12], [13]. It is therefore uncertain how conservation methods perform with respect to sensitivity, though it has been suggested that they are more sensitive than PWM scanning at detecting binding sites with weak affinity for the TF [14]. Here, we test if there are differences between conservation-based methods and PWM scanning on weak and strong binding sites and find the difference in performance to be greater on the stronger binding sites. Thus, contrary to previous reports, our results indicate that conservation-based methods are better at predicting strong than weak binding sites.

## Methods

### Creating a ChIP-seq based benchmark for TFBS predictors

Our primary goal was to create a performance test which would be used to rank several methods for predicting transcription factor binding sites. To do this, we used a set of ChIP-seq peak regions as a “positive” set of binding sites, and larger regions surrounding each peak region as “negative” regions. We reasoned that a good prediction method will score the positive regions higher than the surrounding non-binding regions.

TFBS prediction can roughly be divided into the following two problems: 1) given a region with a known binding site, identify the specific binding site region and 2) identify the genes regulated by a given transcription factor. We therefore made two types of benchmarks based on published transcription factor ChIP-seq data to emulate these problems (Tables 1 and 2). The first, which we refer to as the site benchmark, used all available ChIP-seq peak regions and included a 20,000 bp randomly placed region surrounding each peak. We used 20,000 bp regions to keep the benchmark close to a genome-wide search situation; the regions were roughly 100 and 2000 times larger than the average peak region and TFBS. To avoid making the site benchmark exceedingly difficult and to be better able to compare score distributions in positive and negative regions, we further divided the large negative regions into subregions of 200 bp such that positive and negative regions were of approximately equal length.

**Table 1:** ChIP-seq peak dataset.

TF	Cell-type	Peaks in total	Peaks in promoter set	Avg peak length
NRSF	K562	4329	507 (12%)	154
c-Fos	K562	9781	2697 (28%)	165
c-Jun	K562	12588	1915 (15%)	177
c-Myc	K562	10901	4514 (41%)	184
Max	K562	6688	2564 (38%)	173
GATA1	K562	2548	398 (16%)	180
YY1	K562	3360	2380 (71%)	157
E2F4	K562	8678	5825 (67%)	201
NFKB	GM12878	4555	211 (5%)	223

This table shows the ChIP-seq peak datasets from which the benchmark datasets are generated.

**Table 2:** Motifs used in benchmark.

TF	Motif ID	PWM length	Total info content	Avg info content
NRSF	V$NRSF_Q4	19	13.58	0.71
c-Fos	MA0099.2	7	5.65	0.81
c-Jun	MA0099.2	7	5.65	0.81
c-Myc	V$MYC_Q2	7	6.90	0.99
Max	MA0058.1	10	7.55	0.75
GATA1	MA0036.1	5	4.65	0.93
YY1	V$YY1_01	17	5.17	0.30
E2F4	V$E2F_Q2	6	4.78	0.80
NFKB	MA0105.1	11	9.50	0.86

This table shows the motifs used in the benchmark study. Motifs starting with “V$” are from the Transfac database [23], the others are from Jaspar [22].

The second (promoter) benchmark emulated the problem of mapping TF to target genes. Here, we used RefSeq gene annotations as basis and created two test regions per gene; the first region consisted of 2000 bp upstream and 200 bp downstream of the TSS, whereas the second region was the first intron of the gene. The intron region was limited to maximum 3000 bp. We then mapped the ChIP-seq peak regions to these test regions. The promoter benchmark represents the typical gene-based way of using transcription factor binding site prediction methods, as traditionally the methods are used over relatively small intervals in the vicinity of gene transcription start sites to map their regulatory candidate TFs.

**Figure 1:**
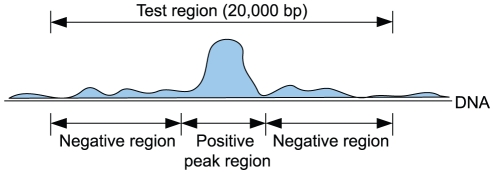
Defining positive and negative regions for the site benchmark. The maximum score in each region is used to calculate the ROC curve. In the site benchmark, the negative regions around a peak are further divided into smaller regions of length 200 bp (not shown). The promoter benchmark is based on the same principle as the site benchmark, but the test regions are then derived from regions surrounding gene transcription start sites and from first introns, and the negative regions are not further divided into smaller regions.

All prediction methods tested in this study output one score per genomic position and DNA strand. This score represents the method's belief that a binding site motif starts at the position. As peak regions represent TFBS, we were interested in testing whether a peak region is scored higher than a non-peak region. To measure the performance of a method, we therefore used the maximum score of all positions inside each ChIP-seq peak region as the score for the peak. Similarly, the maximum scores in the negative region intervals upstream, downstream, or between the peak regions were kept and annotated as scores for the negative regions; see Figure 1.

The peak prediction method we used as basis for our benchmark datasets is a meta-approach that is more strict and specific than the single methods which it is based on [15]. With this additional stringency for peak calling, it could be that the negative regions as defined by this meta approach harbor enriched regions that could possibly represent true binding sites of lesser affinity to the TF. To help avoid any interference of possible false negative binding sites by these “lesser” peaks in the benchmark, we further limited the negative regions by ignoring regions that was defined as a peak by any of the peak calling methods in any of the replicates.

### Defining peaks and promoter regions

The benchmarks were based on public genome-wide ChIP-seq datasets from the ENCODE project [16], available from the UCSC Genome Browser Yale TFBS and HAIB TFBS tracks [17], [18]. The raw tag-count data were then processed by our own peak detection method [15] which we briefly describe here:

ChIP-seq peaks were identified in sample and replicate data by two different peak-finder programs, MACS [19] and SISSRs [20]. Both programs were run using independent background samples to correct for biases in the background tag distribution. To reduce the number of false and spurious peaks identified, only peaks identified by both programs, and in a separate independent replicate sample for MACS, were used in the benchmark. Peak regions were then shortened to 100-400 bp by a peak-trimming procedure to reflect the resolution in ChIP-seq data.

With the peak regions defined, we created the two benchmarks. For the site benchmark we used all available peak data; for each peak we annotated a 20.000 bp region randomly around the peak region as the test region. Any overlapping test regions were iteratively merged by creating a new region containing all of the peaks and with length equal to the sum of the merged regions. This way the ratio between peak and non-peak region were kept constant. The vast majority of test regions only contained one peak region. See Table 1 for an overview of ChIP-seq data used and the peak count for each dataset.

The promoter benchmark was based on the RefSeq gene annotations taken from the RefGene table of the hg18 genome assembly downloaded from the UCSC Genome Browser on October 22, 2009. We only used genes from standard chromosomes (no genes from random or haplotype-specific chromosomes). Two test regions were made from each gene; (1) the promoter region, defined to be 2000 bp upstream and 200 bp downstream of the transcription start site, and (2) the first intron region, which was limited to a maximum length of 3000 bp downstream from the start of the first intron. We only kept one region of any genes that had overlapping promoter or intron regions. Our set of peaks were then mapped to these test regions. If an overlapping peak region was only partially contained within a test region, the test region was extended to fully encompass the peak region. Table 1 shows how many of the total set of genome-wide peaks lie in the promoter and first intron region of a gene and thus were incorporated into the promoter benchmark datasets.

### Performance calculation by ROC score

The prediction methods score each position in the test region on both strands. When calculating prediction performance, we labeled the maximum score inside each ChIP-seq peak region with a positive label and labeled the maximum scores for the negative regions upstream/downstream/between the peak regions with negative labels (Figure 1). After sorting the labels according to descending score, we plotted the receiver operating characteristic (ROC) curve and calculated the area under the curve (AUC or ROC score) [21]. If some regions had the same maximum score, the negatives were counted before the positives. In addition to the ROC curve, we also plotted the ROC-50 curve, where we stopped counting positives after passing 50 negatives.

### Selection of sequence motifs

The motifs were taken from the Jaspar 2009 [22] and Transfac Professional [23] databases. Our only selection bias was that the motifs should have the k-mer sequences available. We did not test several different motifs for each TF. The following motifs were used (given as TF:Motif): NRSF: V$NRSF_Q4, c-Fos and c-Jun: MA0099.2, Max: MA0058.1, GATA1: MA0036.1, YY1: V$YY1_01, E2F4: V$E2F_Q2, NFKB: MA0105.1. See Table 2 for details about the motifs.

### Prediction methods tested in study

We tested our benchmark on five methods, three of which use sequence conservation to improve prediction accuracy.

### PWM search

Position-weight matrices [24] were made from the nucleotide frequency count data in the selected motifs from the Transfac and Jaspar databases together with a standard background model made by counting the number of each nucleotide in the human genome. The PWM was then scored at all positions on both strands.

### MotifScan

The MotifScan method is an alternative to PWM scanning [8]. Instead of making one model for all the known binding sequences (k-mers) of a TF, MotifScan scores a sequence against each k-mer explicitly. The score depends on the number of similar k-mers and the number of differing nucleotides, and in addition a substitution matrix is used for each differing nucleotide. The MotifScan method was implemented by us after the instructions in the original article [8] and the substitution matrix was made from all Transfac and Jaspar sequence motifs. The method was run with original parameter settings.

### Weighted sum

The weighted sum (WS) method is the most straight forward of the methods we tested that use sequence conservation to improve accuracy. The method takes as input the multiple alignment files from UCSC Genome Browser Multiz 28-way alignment. A PWM is used and for each position in the human (hg18) genome, the mouse (mm8) and rat (rn4) genomes (with gaps removed) are also scored in a window reaching 15 bp upstream and 15 bp downstream from the current hg18 position. The maximum score in the window region is multiplied by 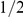 and added to the human score. The method will score motifs conserved in mouse and rat higher than non-conserved motifs.

### Bayesian branch length score

Branch length conservation methods use a phylogenetic tree as input in addition to a sequence alignment to quantify the conservation level of a motif. The total branch length score is defined as the sum of the branch lengths on the phylogenetic subtree spanned by the nodes containing the motif. The Bayesian branch length score (BBLS) [11] differs from the original branch length method [25] in that it weights each branch length by the probability that the branch is under negative selection.

Our implementation of the BBLS is similar to the original; the code for calculating the BBLS score from motif sequence scores was obtained from the authors. 18 placental mammals from the Multiz 28-way multiple alignment was used as input. The BBLS requires a cutoff value where leaf nodes with sequence motif scores above the cutoff are used in the BBLS calculation. We obtained the best cutoff (95 percentile) by calculating the ROC and ROC-50 score on the c-Myc, NRSF and Max datasets using a series of different percentile thresholds.

We introduced a minor novelty, by basically running the BBLS method with MotifScan scores instead of PWM scores as input. The justification for this was that as genomic sequences diverge, the sequence variation span in binding sites under negative selection might not be fully captured by a PWM, which focuses on the “center” of the nucleotide distribution. Our hypothesis was that the BBLS MS method should better tolerate evolutionary sequence drift and outperform the BBLS PWM method. The same cutoff (95 percentile) turned out to be the best and was also used for the BBLS MS method.

### Method implementation

All methods were implemented to be run on a supercomputer cluster, in order to speed up motif search. Python was primarily used, with some extensions written in C for the PWM scanning. The parallell implementation was not strictly necessary, but made it much more feasible to run the methods on all the relatively large datasets.

### Availability

The benchmarks are available as Dataset S2 and S3.

## Results

### Conservation generally improves binding site predictions

Previous studies have shown that information about sequence conservation can improve transcription factor binding site predictions [3], [11], but we wanted to better understand how conservation improves accuracy. We therefore compared standard PWM scanning with two conservation-based methods: the first, a simple conservation method that used the weighted average of the PWM score in homologous regions in the mouse, rat, and human genomes (weighted sum; WS); the second, the more elaborate Bayesian branch length method (BBLS) [11]. In addition, we wanted to test an alternative k-mer-based motif scoring method (MotifScan [8]) and evaluate if it could be used as a basis for an improved conservation-based method. We therefore also tested MotifScan on its own and within the Bayesian branch length framework.

**Figure 2:**
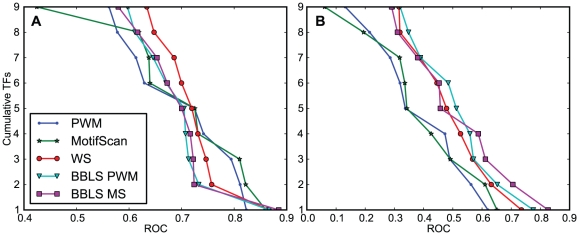
Cumulative ROC score on site and promoter benchmarks. The cumulative number of TF datasets for which a method has a ROC AUC of more than a given value on the **A**) site and **B**) promoter benchmark. Each line represents a method and shows for each point along the y-axis how many datasets that have at least the ROC score given on the x-axis. The ROC score, or area under the ROC curve (AUC), is a measure of accuracy that summarizes the true-positive and false-positive rate and the implied trade-offs at all score thresholds.

Based on previous studies, we expected the conservation-based methods to clearly have superior performance compared to the other methods. However, the results varied more than expected and there were greater differences between benchmarks and scoring methods. On the site benchmark, the MS method had the best median ROC score, followed by PWM. All three of the conservation-based methods were better than PWM and MS on only four of the nine datasets (Fig. 2). Among these methods, the simpler WS method had best median ROC score. This was also the only method that had significantly better ROC score than any other method (p-values 0.0098 and 0.0059 on one-sided Wilcoxon signed-rank test when compared against BBLS PWM and BBLS MS; see Dataset S1 for individual scores and p-values).

The conservation-based methods showed better specificity, however, and the BBLS methods were the only methods that achieved a median ROC-50 score higher than zero (Table 3). Ignoring the results on NRSF, the conservation-based methods were orders of magnitude better, though the absolute ROC-50 scores were still very low. On most datasets, only the conservation-based methods could score some positives higher than the 50 highest scoring negatives; PWM and MotifScan failed to find any positives at this threshold in 7 and 6 TF sets.

**Table 3:** Median and median absolute deviation (MAD) ROC scores on site benchmarks.

Method	Median ROC	MAD ROC	Median ROC-50	MAD ROC-50
PWM	0.7246	0.0955	0.0000	0.0000
MS	0.7251	0.0874	0.0000	0.0000
WS	0.7195	0.0338	0.0000	0.0000
BBLS PWM	0.7047	0.0343	0.0050	0.0031
BBLS MS	0.7001	0.0275	0.0068	0.0041

Although the MotifScan method was better than PWM scanning on five of the nine datasets, the overall differences were not significant (p-value 0.213 on one-sided Wilcoxon signed-rank test). Similarly, the novel MotifScan-based BBLS method, BBLS MS, did generally not outperform the PWM-based BBLS. This result suggests that PWMs may be sufficient to model binding sites for most of the transcription factors in this study, whereas perhaps other transcription factors may require more complex binding site models.

Among the conservation-based methods, it is interesting to note that the simple WS method achieved both higher median and average ROC score than the BBLS methods. Weighted sum actually had better ROC score than PWM-based BBLS on seven of the data sets, the difference being significant. The BBLS methods had much better ROC-50 scores though; BBLS MS was better than PWM, MS, and WS (p-values 0.007, 0.092, and 0.007) and BBLS PWM was also better than these three methods, though the differences were borderline significant (all p-values 0.065).

The differences between the methods were greater in the promoter benchmark (Fig. 2, Table 4). The conservation-based methods were significantly better than PWM and MS in all comparisons (see Dataset S1 for p-values). The BBLS PWM method had the best median ROC score, whereas the BBLS MS had a slightly better average ROC score. BBLS PWM was here significantly better than WS (p-value 0.002). Again, the branch length methods also demonstrated superior ability to discern true binding sites from false positives as measured by the stricter ROC-50 score, but the scores were in the same low range as on the site benchmark.

**Table 4:** Median and median absolute deviation (MAD) ROC scores on promoter benchmark.

Method	Median ROC	MAD ROC	Median ROC-50	MAD ROC-50
PWM	0.3373	0.1357	0.0000	0.0000
MS	0.3425	0.1480	0.0000	0.0000
WS	0.4785	0.0907	0.0000	0.0000
BBLS PWM	0.5121	0.1232	0.0074	0.0042
BBLS MS	0.4572	0.1469	0.0063	0.0050

The top performing method varied from one dataset to another. This shows the importance of using multiple datasets for testing to get a fair comparison between methods; for example, WS had a better average ROC-50 score than PWM-based BBLS due to the good score on NRSF, but overall, BBLS PWM is slightly better than WS (p-value 0.064).

### Conservation has most effect on short and information-poor motifs

**Figure 3:**
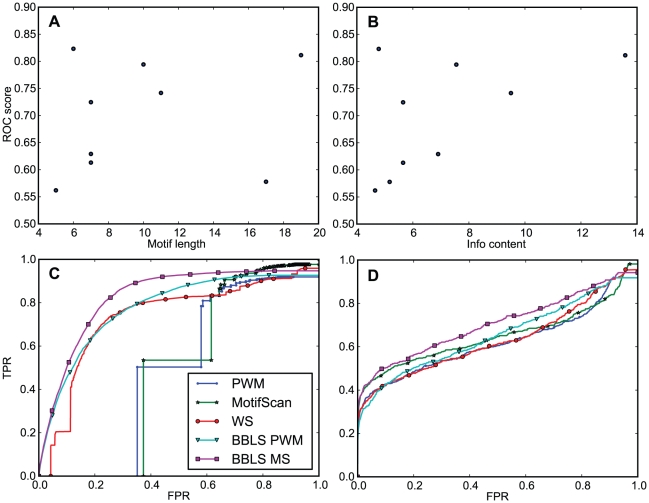
ROC score correlates with motif length and information content. **A**) ROC score for PWM scanning as a function of motif length. **B**) ROC score for PWM scanning as a function of motif information content. Longer, information-rich motif achieve better scores. Note that YY1 has the second longest motif (V$YY1_01), but this motif also has the second lowest information content, which likely explains its lower score compared to the most information rich motif (V$NRSF_Q4). **C**) ROC curves for all methods on the E2F4 dataset in the promoter benchmark. The V$E2F_Q2 motif is one of the least informative motifs and the performance of the prediction methods on the E2F4 dataset is relatively low. **D**) ROC curves for all methods on the NRSF dataset in the promoter benchmark. The V$NRSF_Q4 motif is the most informative motif and the NRSF dataset is among the highest scoring datasets.

The differences between conservation-based methods and pure motif-based methods varied between different TFs and between the two benchmarks. There were some notable exceptions, such as NRSF where the motif-based methods showed better or comparable performance. We noted that NRSF was the best scored dataset overall and also had the longest motif, and therefore hypothesized that PWM scanning performance was generally related to motif length or the motif's specificity. Indeed, PWM ROC scores on the site benchmark were correlated with motif length (Spearman correlation coefficient 0.24) and even more strongly correlated with motif information content [24] (0.49) (Fig. 3).

**Figure 4:**
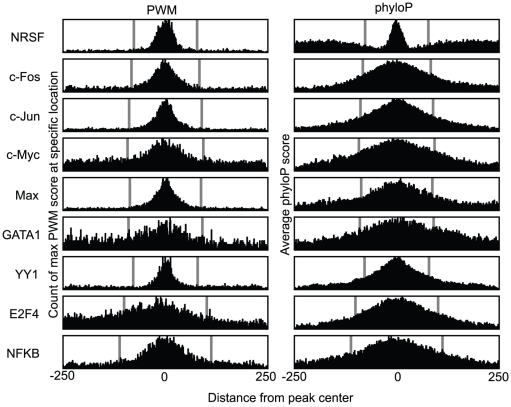
Max PWM score and phyloP values correlate with center of peak regions. The figures show a region of 500 bp surrounding each peak region. On the left is shown for each of the 500 positions the number of times that position has the maximum PWM score in the 500 bp region. On the right is the average phyloP score. The grey lines show the average peak width. Both max PWM score and higher phyloP values tend to be clustered in the center of the peak regions, but the clustering varies for each TF.

We also found that the difference in ROC score between PWM and BBLS PWM correlated with motif information content (Spearman 0.62, two-sided p-value 0.075), meaning that the differences between the conservation-based BBLS PWM and the simpler PWM method were smallest for the information-rich motifs. The four TFs with the largest score difference on the promoter benchmark where either short motifs (c-Myc, GATA-1, E2F4) or had on average low information content (YY1). The short motifs for c-Myc, GATA-1, and E2F4 were also the motifs that apparently had the highest amount of noise around the peak regions, as these motifs showed the least distinct association with the peak center (Figure 4). The figure also shows that the center of the peak regions, where most of the best scoring motifs are located, had stronger sequence conservation compared to its immediate surroundings.

Considered together with the ROC curves of short motifs such as V$E2F_Q2 in Figure 3C and longer information-rich motifs such as V$NRSF_Q4 in Figure 3D, this explains when the benefit of using conservation is greatest; namely when the motif does not contain enough information to distinguish between the random high-scoring sequences and the real binding sites. The conservation methods will less often achieve high motif scores for all aligned genomes in the same loci, and this conservation filtering can therefore make them better at distinguishing between functional (and conserved) motifs and false positives, compared to PWM scanning.

### A complex motif model has most effect on diverse and information-poor motifs

In the comparisons, MotifScan was better than PWM scan on six of nine site datasets and four of nine promoter datasets. One of the TFs with the largest score difference was YY1 where MotifScan had a 20% better ROC score on the promoter dataset compared with PWM scan. The V$YY1_01 sequence motif used on the YY1 dataset is long, but has a low information content with much sequence variation outside of the core of the motif. For example, the average Hamming distance between the YY1 k-mers is 10.3 whereas the average among the other motifs in our benchmark is a mere 2.6.

In theory, MotifScan should be better on motifs with low information content compared to PWMs as its motif representation and scoring is better than a PWM when the binding k-mer-sequences have a lot of variation. In accordance with this hypothesis, we found that the difference between the MotifScan and PWM ROC scores on the site benchmark was negatively correlated with average motif information content (information content divided by motif length; Spearman correlation coefficient -0.69; p-value 0.03 with two-sided corr. test) and positively correlated with k-mer diversity as measured by average k-mer Hamming distance (correlation coefficient 0.64; p-value 0.061); see data in Dataset S1. Thus, MotifScan can give better predictions than PWM scanning for factors where a simple PWM model would “average out” the sequence variation in the k-mers bound by the factor. This likely explains the large difference between PWM and MotifScan on the YY1 dataset.

### Conservation has more effect on detecting strong than weak sites

Others working on assessing performance of prediction methods in the yeast model organism have previously hypothesized that conservation-based methods might be more sensitive than PWM scanning and better at detecting motifs of binding sites that have low affinity for the transcription factor [14]. To test this hypothesis, we divided the ChIP-seq peaks into sets according to peak tag count. In one set, we kept only the sites with peak height higher than the 90 percentile and in another set we kept the sites with peak height lower than the 10 percentile.

**Figure 5:**
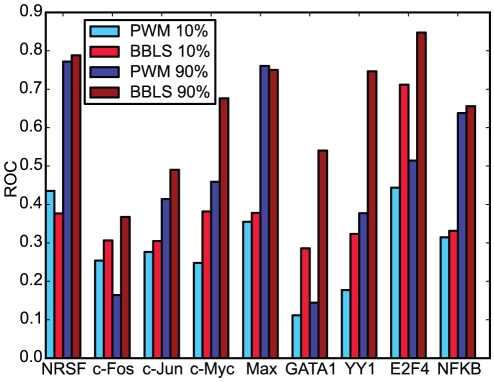
ROC scores for PWM and BBLS PWM on low and high peaks. ROC scores on each TF promoter dataset for PWM and BBLS PWM methods on the lowest peaks (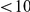 percentile), and highest peaks (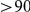 percentile). The difference between PWM and the conservation-based BBLS PWM method is generally greater, and in favor of BBLS PWM, on the higher peaks more than the lower peaks.

Figure 5 shows the ROC scores on the promoter benchmark for PWM and BBLS PWM when the ROC curve is recalculated after only keeping the highest or lowest peaks. For all datasets except c-Fos, sub-datasets consisting of high peaks have better scores than sub-datasets of low peaks. This means that higher peaks correlate better with the sequence motifs than lower peaks and this is consistent with the high peaks being strong binding sites.

According to the previous hypothesis in yeast, we expected the difference in scores between conservation-based methods and PWM scan to be bigger on the low peaks as these represent weak binding events. On the contrary, as seen in Figure 5, the difference in scores between BBLS PWM and simple PWM scanning was generally greater on the high peaks. Except for Max, all the datasets tested show greater score differences in favor of BBLS PWM on higher peaks (above 90 percentile) than on lower peaks (below 10 percentile); see Dataset S1. For NRSF, the PWM performs better than the conservation-based BBLS PWM on the lower peaks.

**Figure 6:**
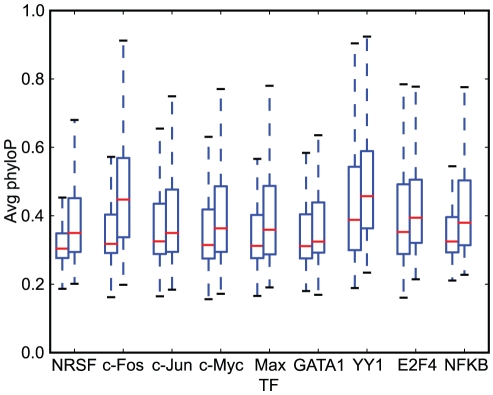
Distribution of phyloP scores in lowest and highest peaks. Boxplot showing for each TF the averaged phyloP scores in promoter peak regions on lowest peaks (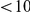 percentile), and highest peaks (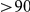 percentile). The higher peaks generally show higher sequence conservation across genomes.

One explanation for this result could be that the motifs in the higher peaks are more conserved than in the lower peaks. Indeed, by looking at average phyloP conservation scores [26] in the low and high promoter peak regions of all TFs, we found that high peak regions have 7.5% higher conservation scores than low peaks; see also Figure 6. Considering how short the sequence motif is compared to the whole peak region, this is a significant difference, and contributes to explaining the difference in scores between PWM and BBLS PWM methods.

This result shows that strong binding sites are generally more highly conserved than weak binding sites are. Consequently, although including conservation information can help in detecting weak binding sites, conservation information does apparently not give an increased sensitivity at recognizing weak sites, but an increased specificity at recognizing strong sites.

### ChIP-seq data from another cell-line validates the benchmark results

The binding of a TF to DNA depends on many factors, which will vary from one context to another. This can, for example, yield differences in binding sites between different cell-types. To test whether our benchmark results were cell-type dependent, we ran the same peak detection method on datasets from the HeLa-S3 cell line for TFs c-Myc, c-Fos, E2F4, and Max and created additional site datasets on which we ran all of the methods. The results using HeLa-S3 data mainly showed the same trend as the original results obtained using the K562 data in the benchmark (see Dataset S1), but with some exceptions. c-Fos was scored higher in HeLa-S3 than in K562 by all methods, with PWM and MS giving best results. For the other datasets, the scores in HeLa-S3 were in the same range as for K562, and expect for Max, the method ranks were mostly similar. Only the conservation-based methods had ROC-50 score greater than zero. The overall results suggest that the benchmark gives a fair judgement of the relative performance of the methods, but the score variations again demonstrate the importance of evaluating methods on many different datasets.

## Discussion

In this article we have described a benchmark for testing methods that predict transcription factor binding sites. Our positive set of binding sites is based on ChIP-seq data and computationally predicted ChIP-seq peak regions. Although ChIP-seq is considered state-of-the-art technology for mapping transcription factor binding sites, there are at least four concerns in using such data for creating a fair and unbiased benchmark. First, ChIP-seq is cell-context specific, whereas motif detection is not. Which of the potential binding sites a transcription factor actually binds depends on the state of the cells, whereas computational prediction based on sequence motifs will not have this kind of bias. We assume that any bias due to the cell-context of the ChIP-seq peak regions have the same effect on the performance of the methods tested and only work to reduce the methods' overall performance. Based on our tests with ChIP-seq data from two different cell lines, this assumption seems to hold.

Second, using ChIP-seq data means that we cannot separate between direct and indirect binding. Because a transcription factor can bind via cofactors and without a sequence-specific motif, this indirect binding can introduce false positive peaks that results in more false negatives in the predicted sites of all methods.

Third, a major concern is the quality and correctness of the peak regions. We use ChIP-seq data from the highly standardized ENCODE project [16] so we expect minimal noise in the source data due to differences in experimental procedures between the cell line datasets. Also, our peak detection method has been shown to be highly accurate when tested against other common methods of peak detection [15]. As described in Methods, the set of derived binding sites are not necessarily complete, but are thought to represent the sites with the highest affinity for the transcription factor and should therefore be correlated with TF sequence motifs. In the benchmarks, we removed from consideration any regions of lesser affinity that are predicted to be peaks by MACS or SISSR alone, but that are not called as peaks with our stricter meta-approach. Given that we found similar relative performance between methods when using data from different cell-lines, we believe the benchmark gives a fair ranking of the methods. For now, ChIP-seq is probably the best technique available for genome-wide mapping of transcription factor binding sites in mammals.

Fourth, an issue which complicates performance comparison and which also explains some of the performance difference between the methods tested, is that many PWM models obtain their maximum score so frequently that it becomes impossible to sort the relatively large predicted regions according to score. In our benchmark, we take a conservative approach when calculating the ROC curve and add all negatives prior to adding positives when scores are equal. This favors the conservation-based methods, whose scoring depends on several genomes and therefore less often achieve maximum scores but give more fine-grained predictions compared to for example PWM scanning which is more penalized, especially on the shorter motifs. This can perhaps to some degree explain why conservation-based methods are so much better relative to PWM scanning on the promoter benchmark than they are on the site benchmark.

Another likely reason for the superiority of the conservation-based methods on the promoter benchmark, as compared to the site benchmark, concern the peak regions themselves. The promoter peaks are higher than the non-promoter peaks (on average 2.7 times higher, p-value 0.129 on a one-sided Wilcoxon signed-rank test across TFs), and importantly, the promoter peaks have more conserved sequence as measured by phyloP score (p-value 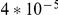). We therefore expect the motifs to be better conserved in the promoter peaks as well.

In sum, we have created comprehensive benchmarks for methods which predict the location of transcription factor binding sites and have used the benchmark to evaluate the effects of using different motif representations and of using comparative genomics in predictions. We found that the methods that use conservation generally achieve better performance than methods that only use a single genome as input, especially on high-affinity binding sites. For good information-rich motifs, however, it might not be necessary or even beneficial to use conservation to predict binding sites.

The benchmarking has shown that the methods for TFBS prediction can and should be improved. As more genomes are made available, comparative genomics approaches, such as the branch length methods and phylogenetic shadowing [27], can be very valuable for improving TFBS prediction. However, given the relatively small performance differences between elaborate and simpler conservation methods in our study, it is likely that new methods also could benefit from integrating more biological data to improve accuracy [28]. We also suspect that the full benefit of more elaborate motif models will be seen as more binding site sequences are made available and incorporated into the motifs.

## Supporting Information

Dataset S1 **ROC scores and results.** Scores pr TF and additional results. The file is in Microsoft Excel format.
(XLS)

Dataset S2 **Site benchmark peak regions.** Genomic loci of the test regions and peak regions for the site benchmark dataset. Each line gives the test region (chromosome, start, stop) and the transcription factor used in the test (either cfoshela, cfos, cjun, cmychela, cmyc, e2f4hela, e2f4, gata1, maxhela, max, nfkb, or nrsf; the “hela” suffix indicates peaks in HeLa-S3), followed by any peak regions therein (if applicable) separated by semi-colons. The peaks predicted by MACS and SISSR in either replicate are available from http://www.bigr.medisin.ntnu.no/data/tfbs-chip-seq-benchmark/macssissr.zip. These are the regions ignored in our benchmark studies, as long as they do not overlap with peaks as given in the benchmark datasets.
(TXT)

Dataset S3 **Promoter benchmark peak regions.** Genomic loci of the test regions and peak regions for the promoter benchmark dataset; see the description of Dataset S2 for details.
(TXT)
